# Nutrient film technique systems for coriander production: A comparison of aquaponics and hydroponics in UAE

**DOI:** 10.1371/journal.pone.0340364

**Published:** 2026-01-20

**Authors:** Ahmed Al Jenaid, Mohamed El Mahi, Abdulla Saeed Bathaqili, Abdallah Khamas Alalawi, Chythra Somanathan Nair, Drishya Nishanth, Radhakrishnan Subramanian, Ramya Manoharan, Abdul Jaleel

**Affiliations:** Department of Integrative Agriculture, College of Agriculture and Veterinary Medicine, United Arab Emirates University, Al Ain, United Arab Emirates; SRM University AP, INDIA

## Abstract

As global populations rise, the need for sustainable agricultural practices becomes increasingly urgent, especially in regions facing water scarcity and harsh environmental conditions. Traditional farming methods are often unsuitable for arid areas, leading to the exploration of alternative techniques like hydroponics and aquaponics. These soilless systems offer efficient water use and nutrient management, making them ideal for regions where conventional farming is challenging. This study compares the efficiency of Nutrient Film Technique (NFT) systems in aquaponics and hydroponics for coriander production. Both systems provide controlled environments for plant growth but differ in their nutrient sources—aquaponics integrates fish farming, while hydroponics relies on synthetic nutrient solutions. The aquaponic system incorporated Nile Tilapia (*Oreochromis niloticus*), which provided nutrients through fish waste, while the hydroponic system used a commercially prepared nutrient solution. Coriander, as a leafy herb, can perform well in aquaponic systems because nitrogen, phosphorus, and some micronutrients from fish waste support growth. Nutrient availability depends on fish stocking density, feed quality, and system maturity, with well-fed fish and a mature system enhancing nutrient release. However, long-term or high-yield production may still require supplementation of elements like calcium, magnesium, or iron, as fish effluent alone may not fully meet crop nutrient demands. Key parameters like plant growth (total weight, shoot length, root length), water quality (pH, nitrate, phosphate), and biochemical attributes (chlorophyll, phenol content, antioxidant activity) were assessed. Results indicated that the aquaponics system outperformed hydroponics in plant growth parameters, with coriander grown in aquaponics showing greater total weight, shoot length, and root length. Aquaponics-grown plants also exhibited higher chlorophyll content and antioxidant activity, suggesting improved photosynthesis and nutritional quality. While hydroponic plants had slightly higher phenolic content, aquaponics facilitated better mineral accumulation, particularly for calcium, magnesium, phosphorus, and zinc. The findings demonstrate that aquaponics offers a more sustainable and efficient approach to coriander production, providing higher yield and superior nutritional quality compared to hydroponics. This highlights the potential of aquaponics for enhancing food security in water-scarce regions.

## 1. Introduction

As the global population continues to grow, the demand for food increases which places more pressure on the agricultural systems. The United Arab Emirates (UAE) faces significant challenges for agriculture due to its harsh climate, characterized by high temperatures and minimal rainfall [1,2]. The country’s arid conditions, limited water resources, and infertile soil make traditional soil-based agriculture unsustainable [1]. Water scarcity is the primary limiting factor for food production, with the agricultural sector consuming over 56% of total water use while contributing less than 1% to GDP [3]. Soil salinity, low agricultural water productivity, and poor soil suitability further constrain local agriculture [3,4]. Sustainable food production is now a critical concern due to the over-exploitation of natural resources and the urgent need to feed a growing population [5]. Key to sustainable agriculture is minimizing water usage and reducing dependency on chemical fertilizers [6]. To address these challenges, alternative approaches such as deficit-irrigation strategies and integrated farming systems are proposed [7,8]. There is also an increasing interest in other sustainable practices like climate smart agriculture. These methods aim to conserve resources, maintain soil fertility, and reduce environmental impact while ensuring agricultural productivity and economic viability [8,9]. Climate-smart agriculture systems like hydroponic and aquaponic systems offer sustainable solutions for food production in water-scarce regions like the UAE. These climate-smart technologies demonstrate significant water saving up to 90% less water than conventional methods [10,11]. These systems require minimal land and can be integrated into urban environments, addressing space limitations in cities [11]. Hydroponic and aquaponic methods also reduce or eliminate the need for agrochemicals, promoting cleaner food production [12]. As climate change impacts intensify, these innovative farming techniques offer promising alternatives for sustainable food security, particularly in arid climates, by maximizing resource utilization and increasing yield per unit area [12,13].

Hydroponics is a soilless system that allows crops to be grown without soil with the help of a nutrient-rich water solution that nourishes plants directly [14]. This technique offers several advantages over conventional agriculture, including higher crop yields, reduced water consumption, and the ability to grow crops in areas with poor soil quality [15,16]. This method can save up to 70–80% of water compared to soil-based farming [17]. Hydroponics encompasses various systems, including active and passive methods, and can utilize different growing mediums such as Rockwool, Hydroton, and Perlite [14]. The technology is particularly beneficial in urban areas with limited space, enabling year-round cultivation regardless of geographical constraints [18]. Hydroponic systems require less water, space, and pesticides than conventional agriculture, making them more environmentally friendly [19]. However, hydroponic systems depend heavily on synthetic fertilizers, raising concerns about their long-term environmental sustainability [20]. To address these concerns, aquaponics has been gaining attention as a more sustainable alternative. Aquaponics refers to a system that combines aquaculture (raising aquatic animals like fish) with hydroponics (cultivating vegetable plants in water) in a symbiotic environment [21]. A recirculatory aquaponic system combines fish farming with plant cultivation in a closed-loop environment. In this system, nutrient-rich waste from fish is recycled as a natural fertilizer for plants, reducing the need for chemical inputs, and making it an environmentally friendly option. It also reduces environmental impacts by minimizing nutrient discharge [22]. Studies also show that it saves up to 90% less water than conventional production methods [10]. The versatility of aquaponics allows for various applications, including urban food production, industrial-scale rural operations, and small-scale farming in developing countries [23]. Although further research is required to evaluate all the potential benefits, aquaponics demonstrates significant potential as a sustainable and efficient food production system.

The Nutrient Film Technique (NFT) is one of the most used types of hydroponic systems due to its efficiency in nutrient delivery and minimal water use [24]. In this system, a thin film of nutrient-rich water flows over the roots of plants. One of its main advantages is its water and nutrient use efficiency, making it ideal for regions with limited water resources, such as arid lands. NFT can save 70–90% of water compared to conventional farming methods [25]. This technique also offers advantages such as space efficiency, economic feasibility, higher yields, and improved crop quality [26]. It also reduces growing time, allows year-round production, and minimizes pest and disease incidence [25]. Recent years have seen increased interest in NFT research, particularly from 2016 to 2019, likely due to its efficiency, ease of handling, productivity gains, and potential for reducing carbon footprints [27]. NFT has shown superior performance compared to geoponics, with significantly higher yields observed in NFT systems [24]. In arid regions, NFT reduces the strain on water resources while maximizing crop production, offering a viable solution for sustainable agriculture. The potential of NFT in aquaponics is significant, as the system can integrate fish farming with plant cultivation, using fish waste as a natural nutrient source. This combination enhances sustainability and resource efficiency, making NFT-based aquaponic systems a promising option for food production in water-scarce environments. According to [28], the survival and productivity of crops in Nutrient Film Technique (NFT) systems vary by species. However, much research has not yet been conducted to determine whether it can match the productivity levels of hydroponics, particularly for widely consumed crops like coriander.

Coriander (*Coriandrum sativum*) is a widely used herb, valued for its culinary and medicinal properties. It is commonly used in various cuisines worldwide, particularly in Middle Eastern, Asian, and Mediterranean dishes, making it a staple in the UAE. Rich in vitamins A, C, and K, as well as antioxidants, coriander plays a significant role in promoting health by aiding digestion and reducing inflammation [29]. Beyond its nutritional benefits, coriander is also relatively easy to grow, making it an ideal candidate for controlled-environment agriculture such as hydroponics and aquaponics. Coriander is a fast-growing, high-demand herb in the UAE, providing an excellent model to test the efficiency of different growing systems. Additionally, its widespread use and economic importance in the region make it a practical crop for studying sustainable agricultural methods. Several studies have explored various factors affecting coriander production in hydroponic systems, highlighting its adaptability but also identifying challenges. Coriander has been successfully grown hydroponically with reduced nutrient concentrations, as [30] found that using only 75% of the nutrients typically recommended for hydroponic lettuce still yielded successful results. Additionally, [31] demonstrated that coriander can thrive in vertical hydroponic systems using LED lighting with specific spectral ratios, enhancing the potential for space-saving cultivation in urban environments. However, the studies also revealed limitations. [32] showed that while coriander can be grown using both fresh and brackish water, heated nutrient solutions lead to reduced yields. Similarly, [33] observed that increased salinity and higher flow rates of nutrient solutions negatively affect growth. Furthermore, [34] found that optimizing seeding density and channel width could improve production outcomes, underscoring the importance of system design. Despite these advances in hydroponics, coriander production in aquaponic systems remains under-researched, particularly in resource-limited or arid regions. Previous aquaponic studies have mainly focused on crops such as lettuce and cucumber in recirculated or mixed cultivation systems [35,36], yet little is known about fast-growing herbs like coriander. Urban farming has been highlighted as a key opportunity to address food security, resource constraints, and spatial limitations in cities, though challenges such as system optimization, policy frameworks, and market access remain [37]. The integration of enviro-typing approaches into aquaponics research may accelerate the development of resilient crops suited to arid environments like the UAE [38]. This experiment aims to compare the performance of NFT hydroponics and aquaponics systems for coriander production in the UAE. The goal is to determine which system offers better plant growth and resource efficiency.

## 2. Methodology

### 2.1. Experimental set up

The research study was done in Falaj Hazza Experimental Farm of the College of Agriculture and Veterinary Medicine, UAEU, located in Al Ain city, 160 km East of Abu Dhabi, the capital city of the United Arab Emirates, at co-ordinate latitude and longitude of 24.2191° N and 55.7146° E, with ambient lighting, average daily temperature of 20.5–28.8°C, and relative humidity of 50%–65%. The experiment was conducted during November 2022- January 2023 using six nutrient film technique (NFT) systems established in a polycarbonate-covered greenhouse.

**Fig 1:**
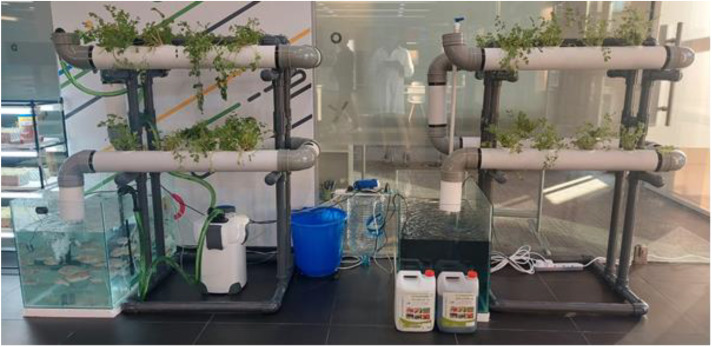
The design of aquaponic and hydroponic systems.

The Nutrient film technique system was made utilizing a vertical model made of PVC pipes (Fig 1). The system was constructed using 4-inch PVC pipes with a total length of 16 meters. The system consisted of 4 lines and 2 rows, with each line being made up of a 2-meter pipe. There was a total of 18 plant cultivation holes, with each hole having a diameter of 5 cm. The system utilized a water pump of power 0.5 hp. A glass tank of 100 liter was utilized for the distinct purpose of storing nutrient solutions. The planting area had a total volume of 50 liters, but it was only half filled, utilizing 25 liters for the nutrient flow. There was a 10-liter biofilter that aided in nutrient cycling and water purification. Altogether, these components sum up to a total system volume of 135 liters. A compact pneumatic compressor system (air pump with a speed of 60 liters per minute) oxygenated the system.

#### 2.1.1 Hydroponic system.

A commercial, water-soluble hydroponic nutrient solution for leafy plants was brought from Max Grow Company, UAE. The solution was applied in two parts: Solution A (26.2% K, 39% Ca, 17.4% Fe, 34%) and Solution B (3.62% P₂O₅, 5.61% Mg, 7.4% S, 0.06% Mn). The final nutrient solution was prepared with 5 mL of Solution A and 5 mL of Solution B per liter of water, ensuring a balanced nutrient supply throughout the cultivation period.

#### 2.1.2 Aquaponic system.

A design similar to the hydroponics unit was used for aquaponics facility. The experimental fish Tilapia (*Oreochromis niloticus*) was procured from the Aquaculture Experimental Station, Falaj Hazza. The introduced fish were daily once fed with the 35% protein floating feeds (from ARASCO feeds) at a rate of 2% of body weight which was updated every month. Proximate composition of fish feed is given in Table 1. Fingerlings of Tilapia fish with an average weight of 52 g were selected and used for the study. No external mineral supplementation was provided. Nutrients were derived exclusively from fish waste and microbial mineralization processes occurring in the system.

**Table 1:** Proximate composition of Fish feed used in the study to feed tilapia.

Moisture (%)	4.78 ± 0.10
Protein (%)	35.21 ± 0.30
Fiber (%)	3.41 ± 0.10
Fat (%)	3.25 ± 0.18
Ash (%)	10.07 ± 0.30
NFE (%)	43.06 ± 0.19

### 2.2 Plant material and growing conditions

High-quality seeds of coriander (Coriandrum sativum L.) were sourced from a local agricultural market. These seeds were sown in pre-moistened Rockwool cubes measuring 2.0 cm × 2.0 cm, which were then placed in plastic containers and watered daily to maintain adequate moisture. Well germinated seedlings after 2 weeks of sowing were transplanted to the growing cups in the Nutrient film technique systems.

### 2.3 Water quality parameters

Water samples from each system were regularly monitored and analyzed on a weekly basis to ensure consistent water quality and system performance. The water quality parameters of temperature, pH, electrical conductivity, total dissolved solids (TDS), NH_3_, NO_3_^-^, NO_2_^_^, and PO₄³ ⁻ were measured using specific instruments. The pH, temperature, TDS and electrical conductivity were measured using a HACH HQD portable meter (Make: HACH; Model: HQ 40d). The concentration of NH_3_, NO_3_^-^, NO_2_^_^, and PO₄³ ⁻ were measured using HACH Multiparameter Colorimeters DR900.

### 2.4 Plant growth parameters

Plant growth parameters are essential metrics used to assess and monitor the development, health, and productivity of plants. Data on plant growth parameters, including root length, shoot length, yield, dry weight, and moisture content, will be collected from both aquaponics and hydroponics systems 60 days after transplanting the seedlings. Plant samples (n = 5) were randomly selected and gently harvested for each treatment per replicate using a spatula. The plants will be placed on tissue paper carefully and remove some of loosely attached media from the roots. The roots will then be thoroughly washed with deionized with minimum root loss of < 3%. After drying with blotting paper, the total lengths of roots, shoots, fresh weight, and other parameters will be determined. Fresh weight will be recorded for each treatment as estimated yield.

### 2.5 Biochemical parameters

#### 2.5.1 Photosynthetic pigments.

Chlorophyll and carotenoid were extracted from the leaves and quantified following the method described by [39]. The results were expressed in milligrams per gram of fresh weight (mg/g FW).

#### 2.5.2 Total Phenol content.

Plants are known to contain a variety of natural antioxidants that protect and preserve their physical and metabolic integrity. For the preparation of methanolic extract, 0.2g of fresh weight plant samples will be weighed and homogenized for 30secs in 20ml of 80% methanol. From the sample mixture, 1.0 ml volume of aliquot was taken, mixed with 0.4ml of hexane to remove the chlorophyll and homogenized for 30secs. The sample will be centrifuged at 5000rpm for 10mins at 25°C. The supernatant will be discarded, and the extract will be washed twice with hexane. The dry weight of the extract will be dissolved in 1ml of 80% methanol (Lester et al., 2013). Plant samples will be analyzed for the total phenolic content using chlorophyll free methanolic extracts by Folin-Ciocalteau (FC) method [40]. 0.5 mL of the sample extract will be mixed with 2.0 mL of 10% FC reagent and left at room temperature for approximately 10 minutes. Following this, 2.0 mL of sodium carbonate (7.5%, w/v) reagent will be introduced and thoroughly mixed. Subsequently, 2.0 mL of distilled water will be added to the mixture, which will then be kept at 40°C for 45 minutes. After incubation, the mixture will be returned to room temperature for 10 minutes. Absorbance readings will be taken at 765 nm using a spectrophotometer after cooling. Gallic acid will be used for the preparation of the standard calibration curve using different concentrations (1mg-10mg/mL). Results will be reported as gallic acid equivalents (GAE).

#### 2.5.3 Antioxidant activity.

To prepare the extract, methanol was added to the powdered plant sample at a ratio of ten parts methanol to one part sample. The mixture was spun overnight, filtered, and the solution was dried in a hot air oven at 50°C for 24 hours. The dried extract was dissolved in 10% dimethyl sulfoxide and stored for future analysis. The ABTS (2,2-azino-bis (3-ethylbenzothiazoline-6-sulfonic acid)) radical scavenging activity was measured following the method of [41]. The ABTS+ solution was prepared by mixing 7 mM ABTS with 140 mM potassium persulfate and incubating in the dark for 16 hours. The solution was diluted with ethanol to achieve an absorbance of 0.70 ± 0.02 at 734 nm. To measure the scavenging activity, 10 µL of extract and 290 µL of the ABTS+ solution were added to a 96-well plate and incubated at 25°C for 6 minutes. Absorbance was measured at 734 nm. The results were then expressed as % inhibition or µmol TE/g of sample as described by [42].

The DPPH (2,2-diphenyl-1-picrylhydrazyl) radical scavenging assay was performed according to the method of [43]. Stock DPPH solutions were made by dissolving 24 mg of DPPH in 100 mL methanol and sonicated for 20 minutes. The working solution was prepared by diluting 20 mL of the stock solution with 80 mL methanol (5X dilution). A standard curve was prepared using Trolox (1 mM), which was also linear between 0 and 800 µM.

### 2.6 Proximate analysis

Proximate analysis of the powdered leaves included estimation of moisture content, ash content, crude fiber, crude fat and protein content, whereas total carbohydrate was calculated using the Eqn., total carbohydrate = 100 – (ash % + moisture % + crude fiber % + crude protein %). The nutritive value of the leaf was expressed in kilocalories/100 g of dry weight of leaves, which was calculated using the formula, nutritive value = (4 × % protein) + (9 × % crude fat) + (4 × % total carbohydrate) [44].

### 2.7 Elemental analysis

The simultaneous determination of major elements and trace metals in plant samples was carried out using Inductively Coupled Plasma Optical Emission Spectroscopy (ICP-OES). Calibration curves for each element were constructed by aspirating mixed calibration standard solutions (Merck). Calibration check standard solutions (Romil) were also aspirated to verify analytical accuracy. Prior to analysis, dried plant samples were acid-digested, and the resulting sample solutions were aspirated into the ICP-OES, and their concentrations were determined from the calibration curves. The macronutrient concentrations (P, K, Ca, Mg) are expressed in mg per gram of dry weight, while the micronutrient concentrations (Cu, Fe, Mn, Na, Zn) are expressed in mg per 100 grams of dry weight.

For ash content analysis, 0.5 g of the dried sample was weighed and placed in a crucible. The crucible with the sample was then heated in a furnace at 550°C until fully ignited. The ash content was calculated by recording the weight difference before and after ignition.

### 2.8 Statistical analysis

An independent t-test was conducted to compare the means of the measured parameters between the aquaponics and hydroponics systems for coriander production. The experiment was replicated three times. The t-test was used to determine whether there were statistically significant differences in plant growth, biochemical parameters and elemental content between the two systems. The analysis was performed using IBM SPSS Statistics software (Version 29). The data were tested for normality using the Shapiro-Wilk test, and homogeneity of variances was assessed using Levene’s test.

## 3. Results

### 3.1 Water quality parameters

**Fig 2:**
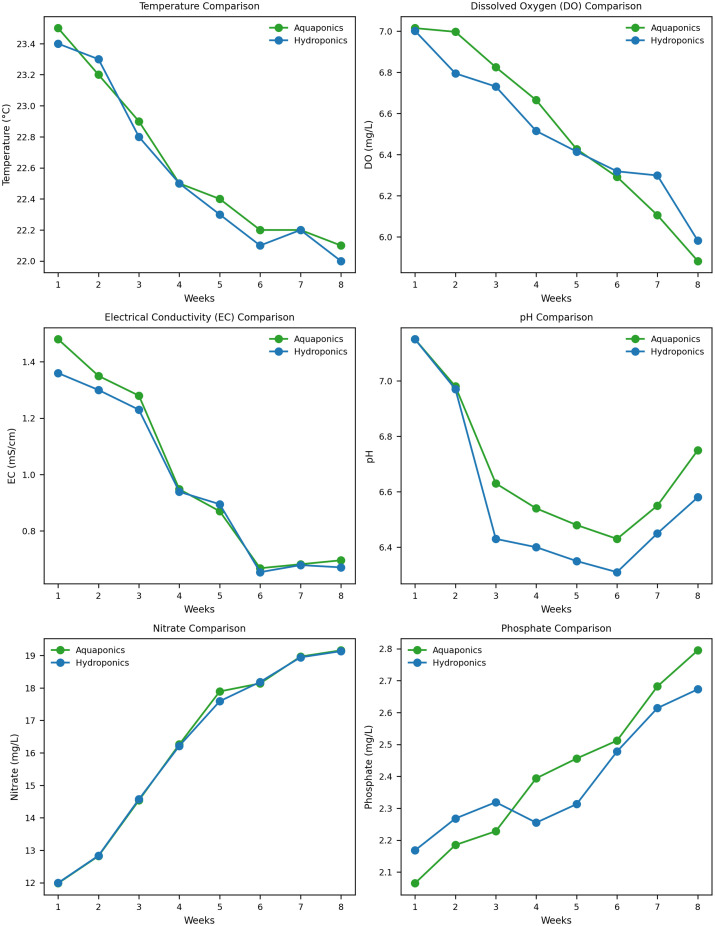
Comparison of Aquaponics and Hydroponics Nutrient Film Technique (NFT) Systems Over Eight Weeks. The graphs present key water quality parameters—Temperature (°C), **Dissolved Oxygen** (**DO**) (mg/L), **Electrical Conductivity** (**EC**) (mS/cm), pH, Nitrate (mg/L), and Phosphate (mg/L)—monitored weekly in both systems.

The data presented in Fig 2 represent the regular monitoring of essential water quality parameters in Aquaponics and Hydroponics NFT systems over an eight-week period. Key indicators such as temperature, dissolved oxygen, electrical conductivity, pH, nitrate, and phosphate were regularly recorded on a weekly basis. These parameters are critical for assessing the efficiency and sustainability of each system in maintaining optimal growing conditions.

#### 3.1.1 Water temperature.

Throughout the 8-week experiment, the water temperature in both systems remained stable, with only slight fluctuations. In the aquaponics system, the temperature decreased from 23.5 ± 0.06°C at the start to 22.1 ± 0.15°C by the final week. A similar trend was observed in the hydroponics system, where the temperature went from 23.4 ± 0.03°C to 22.0 ± 0.05°C by week 8.

#### 3.1.2 Dissolved oxygen (DO).

Dissolved oxygen levels steadily declined in both setups. In the aquaponics system, DO started at 7.015 ± 0.06 mg/L and gradually dropped to 5.883 ± 0.05 mg/L by the end of the experiment. The hydroponics system followed a similar pattern, beginning at 7.002 ± 0.05 mg/L and reaching 5.982 ± 0.07 mg/L by week 8. A slightly greater reduction in DO was observed in the aquaponic system.

#### 3.1.3 Electrical conductivity (EC).

The EC values in both systems showed a downward trend as nutrients were absorbed by the plants. In aquaponics, EC fell from 1.48 ± 0.02 mS/cm in week 1 to 0.696 ± 0.04 mS/cm by week 8. Hydroponics exhibited a similar decrease, from 1.36 ± 0.01 mS/cm to 0.671 ± 0.03 mS/cm over the same period. This reduction indicates effective nutrient uptake in both systems, with a slightly steeper decline in aquaponics.

#### 3.1.4 pH levels.

pH measurements showed gradual shifts across both systems, though both remained within suitable ranges for plant growth. In aquaponics, pH started at 7.15 ± 0.05 and reduced to 6.75 ± 0.08 by the final week. Meanwhile, hydroponics began with a pH of 7.15 ± 0.02, which lowered to 6.58 ± 0.08. The pH drop was slightly more pronounced in the hydroponics system.

#### 3.1.5 Nitrate concentration.

Nitrate levels showed a consistent rise in both systems. In aquaponics, nitrate increased from 11.984 ± 1.25 mg/L in week 1 to 19.158 ± 2.83 mg/L by week 8. The hydroponics system also saw a comparable increase, from 11.995 ± 1.55 mg/L to 19.132 ± 1.22 mg/L over the same time.

#### 3.1.6 Phosphate concentration.

Phosphate levels followed an upward trend over the 8 weeks. In the aquaponics system, phosphate concentration increased from 2.065 ± 0.33 mg/L to 2.795 ± 1.10 mg/L. Similarly, the hydroponics system saw an increase from 2.168 ± 0.85 mg/L to 2.673 ± 2.58 mg/L.

### 3.2 Plant growth parameters

**Fig 3:**
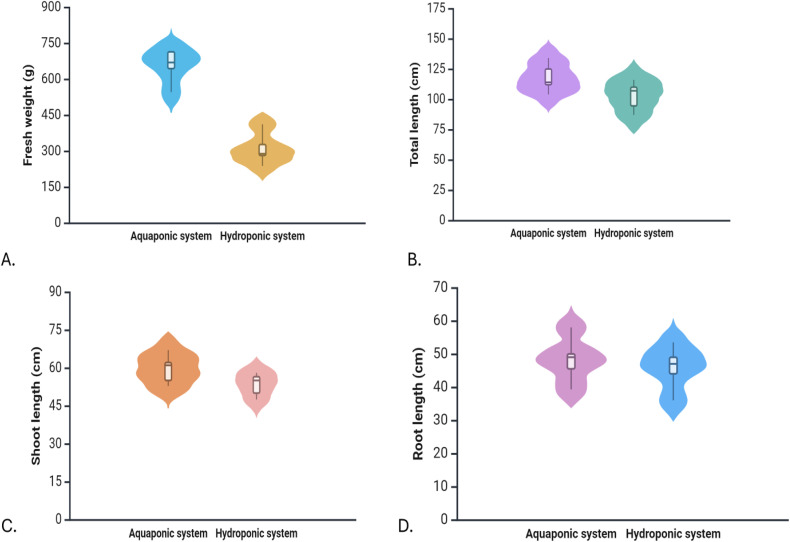
Violin plots comparing plant growth parameters between Aquaponic and Hydroponic systems. (A) Fresh weight (g), (B) Total length (cm), (C) Shoot length (cm), and (D) Root length (cm).

Different growth parameters were regularly monitored and recorded to compare the performance of plants grown in Aquaponic and Hydroponic systems. The distribution of key growth metrics, including fresh weight (g), total length (cm), shoot length (cm), and root length (cm), across both systems are given in Fig 3. In this experiment, aquaponics-grown plants exhibited significantly higher total weight, longer total length, and longer shoot and root lengths compared to hydroponics-grown plants. On average, the total weight of plants in the aquaponics system was 657.34 g which was more than double the average weight of 309.34 g observed in the hydroponics system. Similarly, plants in the aquaponics system exhibited greater total length, with an average of 117.80 cm as compared to 102.90 cm in the hydroponics system. Shoot length also followed the same trend, with aquaponics-grown plants reaching 59.58 cm on average, while those in the hydroponics system averaged 53.40 cm. Root length also showed a slight but consistent advantage in aquaponics, with an average of 48.36 cm, compared to 45.90 cm in the hydroponics system. These results indicate that the aquaponics system provided a more favorable environment for coriander growth, resulting in greater biomass accumulation, taller plants, and longer root systems.

### 3.3 Biochemical parameters

#### 3.3.1 Photosynthetic pigments.

**Fig 4:**
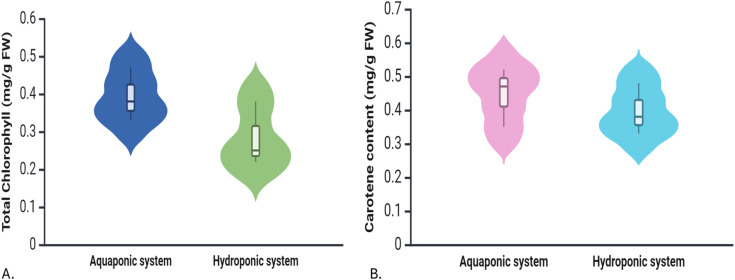
Violin plots comparing photosynthetic pigment concentrations between Aquaponic and Hydroponic systems. (A) Total chlorophyll content (mg/g fresh weight), and (B) Carotene content (mg/g fresh weight).

The total chlorophyll content and carotene content (shown in Fig 4) was studied for coriander plants grown in both aquaponics and hydroponic systems. The aquaponic-grown plants had higher chlorophyll content, although not statistically significant. Coriander grown in the aquaponics system had a higher total chlorophyll content (0.39 mg/g FW) compared to the hydroponics system (0.28 mg/g FW). When divided into components, aquaponics-grown plants also showed higher chlorophyll A (0.29 mg/g FW) and chlorophyll B (0.09 mg/g FW) compared to hydroponics-grown plants, which had 0.24 mg/g FW of chlorophyll A and 0.07 mg/g FW of chlorophyll B.

The average carotene content in aquaponics-grown coriander was 0.45 mg/g FW, while hydroponically grown plants had a slightly lower average of 0.40 mg/g FW. This indicates that plants grown in the aquaponics system produced more carotene.

#### 3.3.2 Total phenol content.

**Fig 5:**
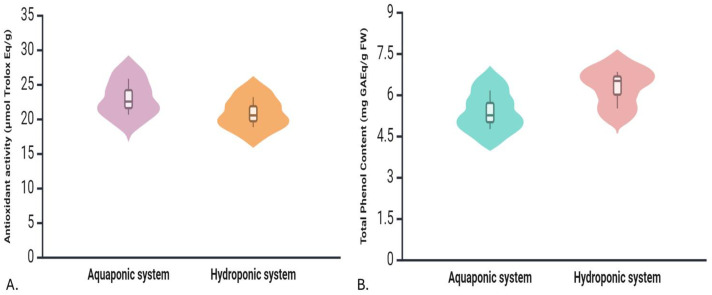
Violin plots comparing antioxidant activity and total phenol content between Aquaponic and Hydroponic systems. (A) Antioxidant activity (µmol Trolox Eq/g) and (B) Total phenol content (mg GAEq/g fresh weight).

From the results (given in Fig 5), it was observed that hydroponically grown coriander exhibited a higher average total phenolic content, with plants accumulating 6.28 mg GAEq/g FW, compared to 5.38 mg GAEq/g FW in the aquaponics system. The difference was not statistically significant.

#### 3.3.3 Antioxidant activity.

In this experiment, antioxidant activity, measured through DPPH radical scavenging ability, was higher in aquaponics-grown coriander (data given in Fig 5). The average antioxidant activity was 22.97 μmol Trolox equivalent/g FW in aquaponics, compared to 20.82 μmol Trolox equivalent/g FW in hydroponics. The difference is not statistically significant.

### 3.4 Proximate analysis

Coriander grown in aquaponics systems demonstrated superior nutritional characteristics, particularly in terms of protein, fat, and moisture content, compared to hydroponically grown plants.

The ash content, which reflects the mineral composition, was relatively similar between both systems, with aquaponics showing an average of 1.67% and hydroponics 1.60%, indicating consistent mineral uptake across both setups. Protein content was notably higher in aquaponics-grown coriander (3.10%) compared to hydroponics-grown plants (2.17%), pointing to better nitrogen utilization in the aquaponics system. Fat content was also slightly higher in aquaponics, with an average of 3.80%, compared to 3.50% in hydroponics. Similarly, the fiber content was marginally greater in aquaponics-grown coriander (6.13%) compared to hydroponically grown coriander (5.87%).

### 3.5 Elemental analysis

**Fig 6:**
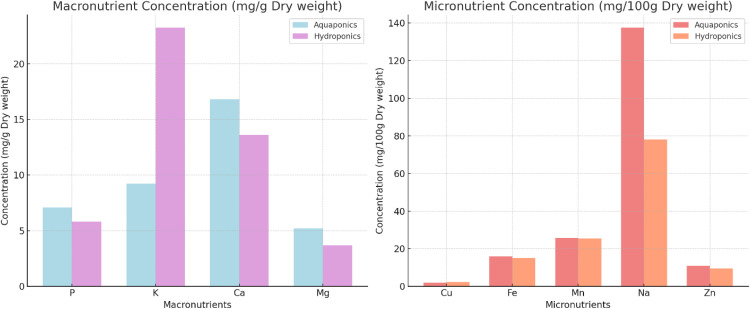
Comparison of nutrient concentrations between Aquaponic and Hydroponic systems. Nutrients analyzed include Calcium (Ca), Copper (Cu), Iron (Fe), Potassium (K), Magnesium (Mg), Manganese (Mn), Sodium (Na), Phosphorus (P), and Zinc (Zn).

The mineral content of coriander grown in aquaponics (A) and hydroponics (H) systems was analyzed to determine levels of key nutrients such as calcium (Ca), copper (Cu), iron (Fe), potassium (K), magnesium (Mg), manganese (Mn), sodium (Na), phosphorus (P), and zinc (Zn) (given in Fig 6). Coriander grown in aquaponics generally showed higher mineral content across most measured nutrients, particularly calcium, magnesium, phosphorus, and zinc, compared to those grown in hydroponics.

Aquaponic-grown coriander exhibited a higher average calcium content (16.80 mg/g DW) compared to hydroponics-grown plants (13.61 mg/g DW). Similarly, magnesium levels were higher in aquaponics (5.22 mg/g DW) than in hydroponics (3.68 mg/g DW).

Phosphorus content also followed a similar trend, with aquaponics-grown plants showing an average of 7.09 mg/g DW, while hydroponically grown plants had 5.83 mg/g DW. Zinc levels were greater in aquaponics, averaging 10.97 mg/100g DW, compared to 9.56 mg/100g DW in hydroponics. Sodium levels were more pronounced in aquaponics (137.55 mg/100g DW) than in hydroponics (78 mg/100g DW).

While the differences in manganese content were minimal, copper concentrations were slightly lower in aquaponics-grown plants ( 1.86 mg/100 g DW ) compared to hydroponics-grown plants ( 2.29 mg/100 g DW.

Iron levels were relatively similar between the two systems, with aquaponics-grown plants averaging 15.8 mg/100g DW and hydroponics-grown plants averaging 15 mg/100g DW. Copper levels were slightly lower in aquaponics-grown plants at 1.86 mg/100g DW compared to 2.29 mg/100g DW in hydroponics. Potassium levels were also significantly lower in aquaponics grown plants (9.22 mg/g DW) as compared to hydroponic grown plants (23.22 mg/g DW).

## 4. Discussion

### 4.1 Water quality parameters

The results of the 8-week experiment demonstrated that water temperature remained stable in both the aquaponics and hydroponics systems, with only minor fluctuations. In the aquaponics system, the temperature decreased slightly from 23.5°C to 22.1°C, and a similar trend was observed in the hydroponics system. Maintaining stable water temperature is crucial for both fish welfare and plant growth, as noted by [45]. While minor temperature declines were observed, these remained within acceptable ranges for optimal system performance. In colder conditions, thermal supplementation may be necessary to prevent further temperature losses and maintain favorable conditions for organisms [45]. Additionally, previous studies indicate that elevated temperatures can enhance fish growth without negatively affecting plant yield, emphasizing the importance of thermal control in aquaponic systems [46]. The stability observed in this experiment aligns with research emphasizing water quality as a key factor in aquaponics, directly influencing both fish health and plant nutrient availability [47]. Temperature balance is also important for the maintenance of a balanced microbial community,as nitrifying bacteria such as *Nitrosomonas* and *Nitrobacter* perform optimally in the 25–30°C temperature range [48].

Dissolved oxygen (DO) levels showed a steady decline in both the aquaponics and hydroponics systems throughout the experiment. Although both systems experienced similar patterns, the aquaponics system exhibited a slightly greater reduction in DO. The slightly greater reduction in the aquaponics system can be attributed to the higher biological oxygen demand, as the presence of fish, bacteria, and plants requires more oxygen compared to the hydroponics system alone [49]. Oxygen depletion in aquaponics results from fish respiration and microbial activity, particularly nitrification, which is crucial for nutrient cycling [49]. Studies have shown that using air pumps can effectively increase DO levels, improving plant biomass production in aquaponic systems [50]. Moreover, implementing automated control systems to regulate DO and pH can help maintain optimal conditions for both fish and plant growth, enhancing overall system performance [51].

The Electrical Conductivity (EC) values in both the aquaponic and hydroponic systems showed a clear downward trend throughout the 8-week experiment, as plants absorbed available nutrients. This decline in EC reflects effective nutrient uptake by plants in both systems, with a slightly steeper decrease observed in the aquaponics system. The sharper reduction in aquaponics could be attributed to its dynamic nutrient cycling, driven by fish waste and microbial activity. Previous studies have demonstrated that EC typically decreases over time as plants utilize nutrients in both aquaponic and hydroponic systems [52,53]. Although aquaponic systems often have lower initial EC values compared to hydroponics, they can still support comparable crop yields [53]. Optimal EC levels for plant growth vary depending on the crop, with lower EC ranges (1.4–2.0 dS/m) potentially promoting better growth for certain crops like lettuce [54]. Despite the differences in nutrient profiles, the decline in EC across both systems in this experiment indicates that nutrient absorption was efficient, and the lower EC in aquaponics did not appear to hinder overall plant growth.

The pH levels in both the aquaponic and hydroponic systems showed a gradual decline over the 8-weeks. Although the pH drop was slightly more pronounced in the hydroponic system, both systems had maintained pH ranges suitable for plant growth. Effective pH management is essential for nutrient availability and plant health in both aquaponic and hydroponic systems. Research indicates that lower pH levels, typically around 6.0 to 6.5, can improve plant performance and yield without adversely affecting fish health or the nitrification process in aquaponic systems [55]. The effects of pH may vary depending on the crop and system design. In decoupled aquaponic systems, pH adjustments between 5.0 and 7.0 had little impact on cucumber growth and yield, though they influenced nutrient availability [56].

The nitrate levels in both the aquaponic and hydroponic systems showed a consistent increase throughout the experimental period. In the aquaponics system, nitrate concentration rose from 11.984 mg/L in week 1 to 19.158 mg/L by week 8, while in the hydroponic system, nitrate levels increased similarly, from 11.995 mg/L to 19.132 mg/L. This rise in nitrate levels can be attributed to the natural nutrient cycling and accumulation of nitrogenous compounds as plants continue to grow and uptake nutrients. In aquaponics, the increase in nitrate levels is primarily due to the nitrification process, where ammonia excreted by fish and organic matter is converted into nitrate by beneficial nitrifying bacteria such as *Nitrosomonas* and *Nitrobacter* [57,58]. This conversion is critical for ammonia biofiltration and underscores the importance of maintaining an optimal pH range, as nitrification efficiency is significantly influenced by pH levels. The observed rise in nitrate suggests that the system’s biofilter was effectively processing fish waste and converting ammonia into plant-available nitrate, which is essential for healthy plant growth. In the hydroponic system, nitrate levels increased due to the periodic addition of nutrient solutions, which replenished essential nutrients for plants, including nitrogen. Hydroponic troughs play a significant role in the removal of total ammonia nitrogen (TAN), achieving an average efficiency of 60.4% [59]. The comparable nitrate increase in both systems reflects a well-maintained nutrient balance, ensuring that the plants had access to nitrogen, which is vital for leaf and stem development.

The consistent rise in nitrate in both systems is a positive sign, demonstrating effective nutrient cycling in aquaponics and appropriate nutrient management in hydroponics. However, careful monitoring of nitrate levels is essential, as excessive nitrate accumulation could lead to nutrient imbalances that might affect plant health or growth. Both systems had maintained nitrate concentrations within a range that is generally beneficial for most plant species, contributing to sustained plant productivity over the course of the experiment. Furthermore, nitrogen budgeting indicates that, of the nitrogen input via feed, approximately 39.4% is recovered as fish biomass, 34.7% as dissolved inorganic nitrogen, and 7.6% as organic nitrogen [59], highlighting the efficiency of aquaponics in utilizing nutrients effectively.

The increase in phosphate levels observed in both the aquaponics and hydroponics systems over the experimental period aligns with established findings in the field of aquaponics and hydroponics. In the aquaponics system, the increase in phosphate concentration can be attributed to multiple contributing factors, primarily the contribution of fish feed and waste, which are significant sources of phosphorus in these integrated systems. According to [60], phosphorus levels tend to rise substantially during the initial weeks of operation, as organic waste decomposes and releases phosphorus into the water, thereby enhancing nutrient availability for plant uptake. In the hydroponic system. [61] also reported similar results in the comparative study of aquaponics and hydroponics but phosphate levels were slightly higher in hydroponic systems as compared to aquaponic systems.

### 4.2 Growth parameters

The comparison of coriander growth in aquaponics and hydroponics systems revealed significant differences in all measured growth parameters, with aquaponics consistently outperforming hydroponics. The results indicate that the aquaponics system provided a more nutrient-rich environment for plant growth compared to hydroponics, likely due to the fish waste recycling nutrients into the system. This enhanced nutrient availability resulted in greater biomass accumulation, as evidenced by the higher total weight of plants in aquaponics. Research comparing aquaponic and hydroponic systems for plant growth has yielded interesting results for aquaponics. Some studies found similar or enhanced growth performance in aquaponics compared to hydroponics [62,63], while others reported reduced yields in aquaponic systems [22]. According to [64], lettuce and herbs grown in aquaponic systems frequently achieved growth rates that were comparable to or greater than those of hydroponically grown plants. [65] reported that aquaponic solutions enhanced lettuce growth more effectively than hydroponic systems, indicating positive effects from the water recirculated from aquaculture systems. The consistent supply of bioavailable nutrients from fish wastes also contributed to better overall plant growth, as seen in the increased total length and shoot length. Additionally, the slightly longer root length in aquaponics suggests that the system supported more effective root development, improving water and nutrient uptake.

### 4.3 Biochemical parameters

#### 4.3.1 Photosynthetic pigments.

Aquaponic-grown plants had higher levels of total chlorophyll, chlorophyll A, chlorophyll B, and carotene content. This suggests that the nutrient environment in aquaponics may promote better photosynthetic efficiency, likely due to the consistent availability of organic nutrients derived from fish waste. These results suggest that the aquaponics system may provide a more optimal nutrient balance for pigment synthesis. The increased chlorophyll content in aquaponics plants indicates more efficient light absorption and energy conversion in photosynthesis, which is vital for growth and biomass accumulation. The enhanced synthesis of these pigments is primarily linked to the steady supply of nutrients obtained from fish waste [66]. Various studies indicate that plants grown in aquaponic environments display higher concentrations of chlorophyll and carotenoids compared to those cultivated in soil. This increase in pigment levels contributes to greater photosynthetic efficiency and biomass production [67,68]. This may be due to the balanced nutrient availability provided by aquaponics, which helps maintain optimal pigment synthesis. Studies indicate that aquaponically grown plants, such as lettuce and basil, often exhibit enhanced chlorophyll a and b content, leading to improved photosynthetic efficiency. The presence of organic matter and beneficial microorganisms in aquaponic systems can further promote plant health and vigor, contributing to greater chlorophyll synthesis [67,68].

Plants grown in the aquaponics system had higher content of carotene which may enhance their resilience and contribute to better nutritional value. The study by [69] found that aquaponically grown lettuce had higher levels of carotenoids than hydroponically grown counterparts, attributed to the diverse nutrient availability and the presence of beneficial microorganisms in aquaponic systems. However, results can vary depending on specific plant species, nutrient management practices, and system design. Overall, aquaponic systems can provide favorable conditions for increased carotenoid accumulation in plants compared to traditional hydroponic setups.

#### 4.3.2 Total phenol content.

The current study found no significant difference in phenolic content between coriander plants grown in aquaponics and hydroponics, although hydroponically grown coriander exhibited higher total phenolic content. This suggests that hydroponic plants may have experienced more stress due to variations in nutrient availability or environmental conditions, as phenolic compounds are synthesized in response to various abiotic and biotic stresses [70]. Research indicates that in nutrient-rich environments like aquaponics, plants often prioritize growth over phenolic production, consistent with the growth-differentiation balance hypothesis and optimal defense theory [71,72]. However, responses can vary by species; for example, aquaponic cultivation has been linked to increased phenolic production in basil and parsley compared to soil-grown plants [73]. Additionally, lettuce in aquaponics showed variable nutrient uptake and secondary metabolite production based on cultivar varieties displaying higher polyphenol concentrations despite lower nutrient availability [74].

#### 4.3.3 Antioxidant activity.

Antioxidant activity measured via DPPH showed that aquaponics-grown plants had greater free radical scavenging potential. This result suggests that coriander grown in the aquaponics system had a higher capacity to neutralize free radicals. This is indicative of a higher nutritional value, as antioxidants are vital for human health. The elevated antioxidant capacity in aquaponics-grown coriander could be linked to the more natural and balanced nutrient profile in aquaponics, which promotes the synthesis of secondary metabolites such as carotenoids and phenolics that have antioxidant properties. Research has demonstrated that aquaponic basil can exhibit higher antioxidant enzyme activity, possibly due to the more complex nutrient environment in aquaponics, which promotes secondary metabolite production [75]. Similarly, aquaponically grown lettuce exhibited enhanced total antioxidant activity when dried at room temperature [76].

### 4.4 Proximate analysis

The comparative analysis of coriander grown in aquaponics and hydroponics systems revealed distinct differences in various nutritional parameters, including moisture, ash, protein, fat, and fiber content. These differences are likely to result from the unique environmental conditions and nutrient dynamics of each system.

#### 4.4.1 Ash content.

The ash content of plants is an important indicator of the total mineral and inorganic content present in plant tissues. It provides valuable information on the nutritional quality of the plant. In previous studies, the ash content in aquaponic plants was lower than in hydroponic plants due to insufficient Mg and Ca from fish feed [77]. But in this experiment, slightly higher ash content was observed in plants grown in aquaponics as compared to those grown in hydroponics which might be due to the organic nutrient input from fish waste, which offers a more balanced range of trace elements like iron, zinc, and manganese. In aquaponics, nutrient cycling allows for continuous recirculation of these micronutrients, potentially making them more bioavailable and leading to greater mineral accumulation over time compared to the controlled nutrient supply in hydroponics.

#### 4.4.2 Protein content.

A significant difference in protein content was observed between aquaponics-grown coriander and hydroponics-grown plants, likely attributed to the nitrogen dynamics inherent in aquaponic systems. In aquaponics, fish waste serves as a natural nitrogen source, essential for protein synthesis in plants. The continuous cycling of nitrogen from fish waste may provide a more consistent nitrogen supply, promoting higher protein production in plants. In contrast, hydroponics relies on synthetic nutrient solutions that deliver nitrogen in fixed amounts, which may not sustain optimal protein synthesis over time [78,79]. Moreover, studies indicate that nitrogen availability influences the amino acid profile and overall protein content in plants, highlighting the importance of a balanced nutrient supply for maximizing protein levels [80,81]. Therefore, the enhanced nitrogen dynamics in aquaponics could explain the observed higher protein content in coriander grown within this system.

#### 4.4.3 Fat content.

The fat content of coriander was slightly higher in the aquaponics system compared to the hydroponics system. This difference may be linked to the organic nutrients present in the aquaponics system, which may encourage the production of lipid-based compounds that are vital for cell membrane integrity and stress resistance. This organic nutrient source can lead to higher levels of beneficial fatty acids, such as omega-3 and omega-6, compared to the synthetic solutions commonly used in hydroponics [78]. The presence of natural hormones and enzymes in aquaponics, derived from the interaction between plants and fish, may also contribute to enhanced lipid synthesis. Specific plant varieties cultivated in aquaponics tend to accumulate higher fat content due to their metabolic responses to organic inputs [73]. The continuous cycling of nutrients in aquaponic systems also enhances the bioavailability of essential fatty acids, allowing for more effective absorption compared to hydroponic systems, where nutrient supply is more controlled and less dynamic [81].

#### 4.4.4 Fiber content.

Dietary fiber derived from plant cells plays a crucial role in human health and nutrition. It consists of components resistant to enzymatic digestion, including cellulose, hemicellulose, and lignin [82]. Consumption of fiber-rich foods has been associated with reduced incidence of various diseases, including coronary heart disease, diabetes, and certain cancers [83]. Fiber is a structural component in plants, and the presence of organic matter or nutrients like Nitrogen, phosphorus, potassium enhanced the structural integrity of the plant, resulting in higher fiber content [84]. In this experiment, the fiber content was marginally higher in aquaponics-grown coriander compared to hydroponics-grown plants. This slight increase could be a result of better root development and stronger plant structure in aquaponics, due to the more diverse and organic nutrient base available in this system. In hydroponics, while plants receive all essential nutrients, the lack of organic inputs might limit the production of structural components like fiber.

### 4.5 Elemental analysis

Aquaponics-grown coriander demonstrated higher calcium and magnesium levels compared to hydroponics-grown plants. This can be attributed to the natural cycling of nutrients in aquaponics, where organic matter from fish waste provides a more diverse nutrient supply. Both calcium and magnesium are essential for plant structure and photosynthesis, and their higher concentrations in aquaponics indicate improved nutrient availability and absorption. The dynamic nutrient cycling and improved microbial activity present in aquaponic systems can enhance nutrient availability and uptake by plants.

While iron was relatively similar between the two systems, aquaponics-grown plants had slightly higher levels of nutrients. The continuous breakdown of organic matter in aquaponics may allow for better iron absorption, which is essential for chlorophyll synthesis. The enhanced iron absorption may lead to improved photosynthetic efficiency, positively impacting plant growth and biomass production [85]. Potassium levels were lower in aquaponic systems. Potassium is essential for various physiological processes such as osmotic regulation, enzyme activation, and photosynthesis [86]. [22] also observed similar results and the lower potassium levels in aquaponic-grown plants were due to insufficient levels of potassium in fish feed. Unlike hydroponics, where potassium is added directly, aquaponic plants deplete potassium quickly without immediate replenishment.

When comparing aquaponics to hydroponics, aquaponic systems demonstrate higher phosphorus use efficiency (PUE) and lower environmental impacts due to reduced nutrient inputs and losses. Other studies have reported differences in nutrient composition, with aquaponic plants generally exhibiting lower phosphorus levels but higher concentrations of calcium, potassium, and magnesium [53]. However, the findings of this study indicate that aquaponics-grown coriander contained significantly higher phosphorus levels compared to hydroponics-grown plants. Previous studies by [87] also noted that lettuce cultivated in aquaponic systems exhibited higher phosphorus levels. This elevated phosphorus concentration in aquaponic systems might be from the organic nutrient inputs derived from fish waste, which can provide a more stable and accessible supply of this essential nutrient. Phosphorus is critical for root development and energy transfer, further emphasizing the importance of nutrient sourcing in aquaponic systems. Crop selection and nutrient dynamics play a crucial role in optimizing nutrient removal and overall system efficiency [88,89].

Sodium levels were notably higher in aquaponics-grown plants compared to hydroponics-grown plants. Higher sodium levels have been noted in aquaponically grown plants across various studies [22,54,63]. This increased sodium content is likely a result of the accumulation of fish waste within the recirculating water system [22]. While sodium is not typically essential for plant growth, elevated levels in aquaponics may result from the recirculating water system, which could cause salt buildup over time.

Micronutrient levels such as copper, manganese, and zinc were similar in aquaponics-grown coriander. Research indicates that aquaponic systems facilitate nutrient cycling, allowing for a more balanced spectrum of trace elements, including micronutrients essential for plant growth [90]. This contrasts with hydroponics, which often relies on synthetic nutrient solutions that may not provide the same diversity of micronutrients [91]. Therefore, the superior levels of certain micronutrients in aquaponics-grown coriander suggest that these systems could offer nutritional advantages in plant cultivation.

Recent studies have highlighted the differences in mineral content between plants grown in hydroponic and aquaponic systems. For instance, [92] observed that lettuce and curly endive grown in aquaponic systems exhibited varying mineral compositions compared to those grown hydroponically, suggesting that aquaponic systems can influence nutrient accumulation differently. Furthermore, [93] found that lettuce grown in aquaponic systems showed a 39% increase in fresh mass compared to hydroponically grown counterparts, indicating that aquaponic systems can support enhanced plant growth under certain conditions.

## 5. Conclusion

This study aimed to compare the performance of Nutrient Film Technique (NFT) systems for coriander production in aquaponics and hydroponics in the UAE’s arid climate. Given the region’s water scarcity and soil limitations, sustainable agricultural methods like hydroponics and aquaponics offer promising alternatives for enhancing food security. The focus of the study was to evaluate the growth, yield and nutrient content of coriander in both systems. The findings revealed that aquaponics consistently outperformed hydroponics in key growth parameters. Plants grown in aquaponics exhibited higher total weight, longer shoot and root lengths, and greater biomass accumulation, likely due to the continuous nutrient supply from fish waste recycling. Additionally, aquaponics-grown coriander had improved nutritional quality, with higher protein, fat, and fiber content, compared to hydroponics.

The study also showed that aquaponics enhanced photosynthetic pigment production, with higher levels of chlorophyll and carotene, contributing to more efficient photosynthesis. While hydroponically grown plants had higher phenolic content, aquaponics-grown plants demonstrated superior antioxidant activity, enhancing their nutritional value. Elemental analysis indicated that aquaponics facilitated better mineral accumulation, particularly of calcium, magnesium, phosphorus, and zinc. In conclusion, aquaponics proved to be a more sustainable and productive system for coriander cultivation in arid environments, offering better plant growth, yield and improved nutritional outcomes compared to hydroponics. This highlights the potential of aquaponics as a viable solution for addressing food security in water-scarce regions like the UAE.

## Supporting information

S1 Data Data Set. (XLSX)
